# *Dianthus chinensis* L.: The Structural Difference between Vascular Bundles in the Placenta and Ovary Wall Suggests Their Different Origin

**DOI:** 10.3389/fpls.2017.01986

**Published:** 2017-11-30

**Authors:** Xue-Min Guo, Ying-Ying Yu, Lan Bai, Rong-Fu Gao

**Affiliations:** ^1^College of Life Science and Technology, Hebei Normal University of Science and Technology, Qinhuangdao, China; ^2^College of Biological Sciences, China Agricultural University, Beijing, China; ^3^College of Life Science and Technology, Beijing Forestry University, Beijing, China

**Keywords:** angiosperm, placenta, carpel, origin, comparative anatomy, *Dianthus chinensis*

## Abstract

*Dianthus chinensis* is a perennial herbaceous plant with great ornamental, botanical, ecological, and medicinal value. The pistil of *D. chinensis* is composed of two fused carpels with free central placenta and two separate styles. The placenta is a columnar structure extending about two-thirds the length of the maturing fruit, which is typical of the Caryophyllaceous. Traditionally, free central placenta is thought to have evolved from axial placenta by septal disappearance, and axial placenta to have occurred through fusion of conduplicate carpels with marginal placenta. However, the traditional opinion is becoming more and more inconsistent with the new data gained in recent research of angiosperm systematics. To clarify the origin of *D. chinensis* pistil, the present anatomical study was carried out. The results show that the vascular system of placenta is independent to that of the ovary wall in *D. chinensis*. Moreover, in the central part of placenta there are one or two amphicribral bundles, and correspondingly numerous ones in the pistil which supply the ovules/seeds. It is obvious that the central amphicribral bundles in placenta are comparable to the counterparts in branches but not to those in leaves or their derivatives. Therefore, it is reasonable to deduce that the placenta of *D. chinensis* was not derived from conduplicate carpels through fusion of collateral vascular bundles, and actually a floral axis with ovules/seeds laterally adhering. On the contrary, the ovary wall was the lateral appendages of the floral axis. The result of the present study is completely in agreement with Unifying Theory, in which the placenta is taken as an ovule-bearing branch. Except for *D. chinensis*, the similar vascular organization has been observed in placenta of numerous isolated taxa. But till now, it is uncertain that whether this vascular organization pattern is popular in the whole angiosperms or not. More intensive and extensive investigations are needed.

## Introduction

The angiosperms, or flowering plants, one of the major clades of extant seed plants, are the largest group of embryophytes, with at least 260,000 living species classified in 453 families (Angiosperm Phylogeny Group, 2003). Angiosperms are amazingly diverse in their habitats, size, longevity, overall form, chemistry, reproductive morphology, and genome size and organization (Soltis and Soltis, 2004). Given this diversity, angiosperm phylogeny, branded “an abominable mystery” by Darwin (Darwin and Seward, 1903), remains a topic of intense research to date. The structures of plant flowers are usually less affected by their environment than their vegetative parts over the long-term processes of evolution; therefore, the understanding of floral structure makes an important contribution to the discussion of angiosperm phylogeny, and has been studied by systematists in this context (Endress, 1994).

The carpel is an essential feature of angiosperms and understanding its origin is vitally important to improve of our knowledge of angiosperm phylogeny (Tang, 2008). Although the traditional theory of the origin of the carpel, in which the carpel is the basic unit of the angiosperm pistil and evolved by megasporophyll with ovule bearing on its edge (Eames, 1961; Cronquist, 1988), is widely accepted in angiosperm systematics, and is still taught in the classroom, recent progress in the field, based on molecular biology, has refuted the previously accepted hypothesis.

*Arabidopsis thaliana* flower has four-whorl floral organs, whose growth and development are controlled by different gene combinations (Skinner et al., 2004; Mathews and Kramer, 2012). After the gene that controls the development of the ovary wall is knocked out, the ovule can still develop in the branched placenta (Roe et al., 1997), suggesting that the loss of the ovary wall does not affect the development of the placenta and ovule and is independent of the latter two. When the gene of the control placenta is artificially altered, the ovary wall develops normally, but the placenta changes greatly (Wynn et al., 2014). The co-expression of *REV* and *STM* in the placenta primordium is the characteristic of the top of the branch, suggesting that the placenta is essentially a branch. The placenta may have evolved from an originally separate fertile structure that was recruited later onto so-called carpel (Skinner et al., 2004). In *Petunia* and *Oryza*, the ovary walls and the placenta are also controlled by two different sets of genes (Angenent et al., 1995; Li et al., 2011). Functional gene studies of these model plants indicate that the placenta and ovary wall of angiosperms are controlled by different genes, being equivalent to an ovule-bearing shoot and a foliar organ, respectively, which is obviously contrary to the traditional theory, because according to this theory, each part of the conduplicate carpel, including the ovary wall and placenta, has the same genome. Hence, it is necessary to re-examine the traditional doctrine.

The vascular bundle is a strand of conductive tissue extending lengthwise through the stems and roots of higher plants, including the ferns, fern allies, gymnosperms, and angiosperms. The vascular bundle consists of xylem, which conducts water and dissolved mineral substances from the soil to the leaves, and phloem, which conducts dissolved foods, especially sugars, from the leaves to the storage tissues of the stem and root. The structure of vascular bundles varies among different plant groups (Columbia Electronic Encyclopedia, 2016); however, compared with the softer tissues, which may or may not exhibit fusions, reductions, or elaborations, the vascular skeleton of plants is considered to be more resistant to change (Thomson, 1942). Accordingly, vascular anatomy is considered as an indicator of the ancestral condition (Douglas, 1936). The carpellary bundle, as a relatively conservative structure, has extreme significance phylogenetically. The vascular bundle has different origin and structure in the placenta and ovary wall of angiosperm, corresponding to a shoot and a leafy organ. In kiwi fruit with axile placenta, the axile placenta is independent of the surrounding ovary wall, and the vascular bundles in the placenta are amphicribral, collateral at least in the upper part of the ovary wall (Guo et al., 2013). In the flowers of *Magnolia*, the carpel with marginal placenta is composed of two mutually independent primordia (corresponding to the ovary wall and axilla placenta). The ovary wall and placenta respectively have their own independent vascular bundles, and vascular bundles in them are totally different as showed by the amphicribral vascular bundle in the placenta and the collateral ones in the ovary wall (Liu et al., 2014).

*Dianthus chinensis* L. is an important ornamental, botanical, ecological, and medicinal perennial herbaceous plant which is widely cultivated in Europe and Asia. The investigation of propagation (Fu et al., 2008; Wang et al., 2014), cultivation (Yang et al., 2013), physiology (Ding et al., 2013; Dar et al., 2014), biochemistry (Croxdale and Outlaw, 1983; Han et al., 2015), genetic breeding (Wu et al., 2003; Zhu and Sun, 2015), and other features of *D. chinensis* has been extensive. Regarding pistil anatomy, the vascular supply to the ovary and styles was traced and described by Thomson (1942), and the pistil composition, ovule arrangement, and development of placenta, septum, and seeds were investigated by Buell (1952); however, these accounts barely discussed the evolutionary implications of the vascular bundles.

One of the typical characteristics of the *D. chinensis* pistil is its free central placenta. Over the past 130 years, the nature and origin of the free central placenta and carpel has been controversial, and discussed among investigators of the Caryophyllaceae. For example, Lister (1884) put forward that the placenta was carpellary according to detailed developmental studies of floral organs in Alsineae; but Gibbs (1907) considered the placenta axile for it was an upward extension of the axis arising between carpel primordia, with which it secondarily associated. Rocén endorsed that the placenta was carpellary since free central placenta was derived from central placenta through the fusion of carpels and vanish of the septum according to Buell (1952). In the light of vascular anatomy study in Primulaceae, Douglas (1936) also thought the placenta to be carpellary in this family. However, in Eames' opinion (1951), there were not only normally oriented bundles but also inverted ones in the placenta of Portulacaceae and Primulaceae, the two genera of the Caryophyllaceae, so that the placenta was composed of both axile and carpellary tissues, whereas in cases of only inverted bundles were present the placenta was entirely carpellary. In another study it was described that the placenta of Dianthus was presumed to be fused carpel tissue because the central placental vascular strand in Dianthus represented fused ventral carpellary traces (Thomson, 1942).

In the present study, we dissected *D. chinensis* pistils, and conducted a systematic observation of the shapes, types, and structures of the vascular bundles within it, to explore the difference of vascular structure between the plancenta and the ovary wall and the implications of the vascular anatomy of the forming carpel.

## Materials and methods

Flowers in anthesis were collected from a nursery garden (119.17°E, 39.71°N) attached to the College of Horticulture Science and Technology, Hebei Normal University of Science & Technology. Two days after flowering, one flower was photographed and the pistil was isolated from the other parts of the flower, its morphology and structure examined, and the size of each part of 15 pistils was measured using Vernier calipers and expressed as mean ± standard deviation.

The ovary wall was partially or totally removed and the middle ovary transected. Then the pistil shape, the ovary with or without a large part of the ovary wall, the ovary transection, and the papillae of the stigma were observed and photographed using an Olympus ZX16 stereomicroscope with an Olympus Infinity1 digital camera.

Pistils were preserved in a formalin–acetic acid–alcohol mixture, embedded in paraffin, sectioned serially into sections of 8–10 μm thickness, and stained in safranin and fast green. Complete transverse and longitudinal series were examined and photographed under an Olympus BX51 microscope with an Olympus DP72 digital camera.

Pistils were also softened in 2 M NaOH solution in a 65°C water bath for 30–40 min, squashed, stained in safranin, and mounted with glycerol. The prepared slides were photographed under the microscope.

## Results

**Figure 1:**
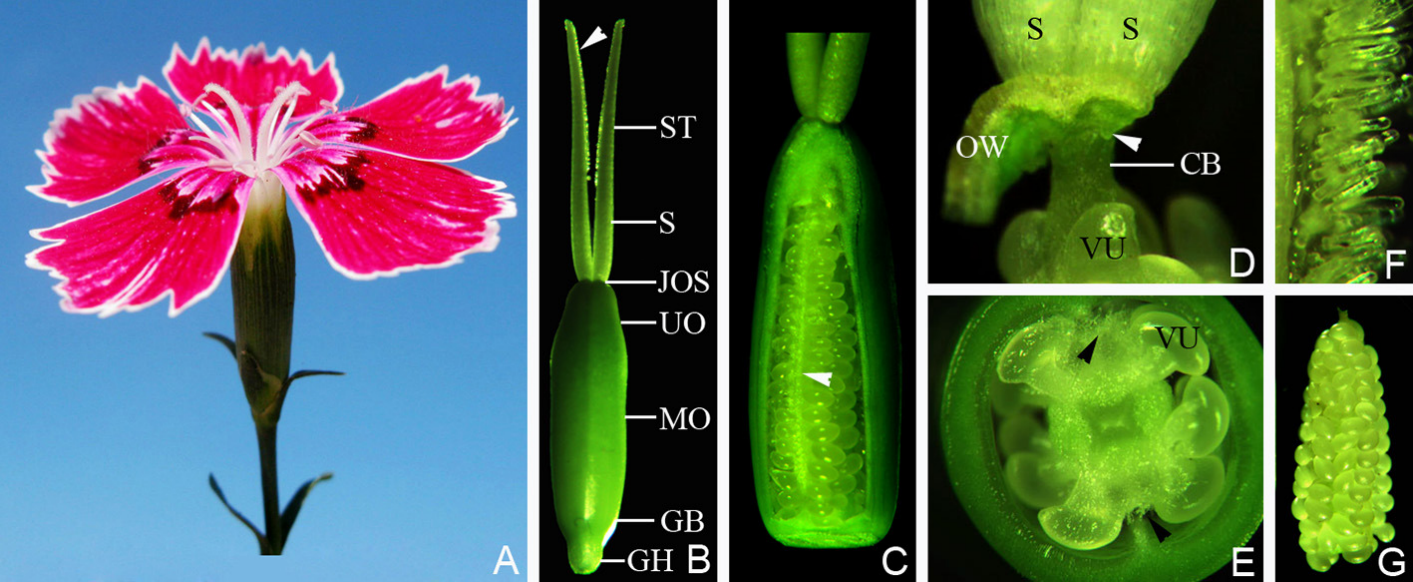
The flower and pistil of *Dianthus chinensis* L. CB, connecting bands between placenta and styles; GB, gynobase; GH, gynophore; JOS, juncture of ovary and styles; MO, middle of ovary; VU, ovule; OW, ovary wall; UO, upper part of ovary; S, style; ST, stigma. **(A)** A flower, showing the upper parts of two separate stigmata. **(B)** The appearance of a pistil 2 days after flowering, showing the shapes of the gynophore, ovary, style, and stigma, and the location of the papillae (arrow). **(C)** A pistil (with part of the ovary wall removed), gynophore, and stigma, showing the central placenta bearing ovules in the ovary and the distribution of papillae covering the transmission tissue (arrow). **(D)** The pistil with the ovary wall removed, showing the lower end of the style (arrow) and connecting bands between the placenta and the styles. **(E)** Transection through the middle of an ovary, showing the papillae distributed at a position corresponding to the septum. **(F)** Part of the stigma, showing the form of the papillae on the abaxial side of the stigma. **(G)** A free central placenta bearing ovules, showing ovule arrangement on the placenta.

**Figure 2:**
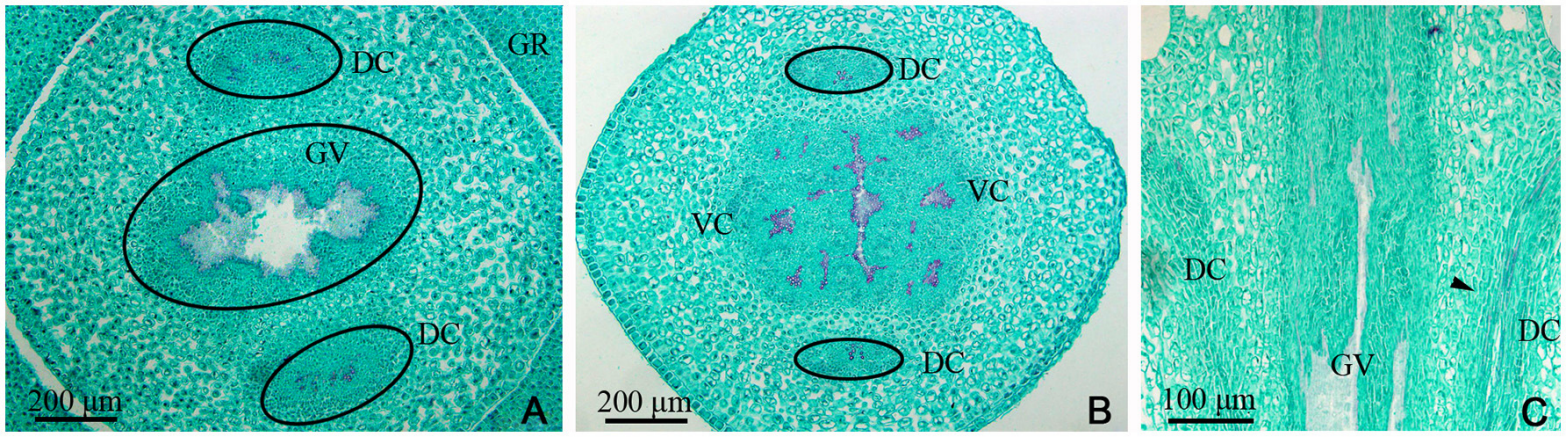
*Dianthus chinensis* L. gynophore sections. DC, dorsal carpellary trace; GR, glandular ring; GV, gynophore vascular bundle; VC, ventral carpellary trace. **(A)** Transection of a gynobase surrounded by a nectary, showing an amphicribral gynophore vascular bundle and the xylem temporarily present in the center of the collateral dorsal carpellary traces branching out of the gynophore vascular bundle. **(B)** Transection a short distance above the level in **(A)**, showing the differentiating ventral and lateral carpellary traces from the gynophore vascular bundle and differentiated collateral dorsal carpellary traces (circle). **(C)** Longitudinal section of a gynophore, showing two differentiating dorsal carpellary traces from the gynophore vascular bundle and the transformation from an amphicribral to a collateral bundle observed ascending the gynophore (arrow).

The *D. chinensis* pistil consisted of four regions: gynophore, ovary, style, and stigma, that merged with one another. The gynophore was (3.08 ± 0.38) mm long, with the lower part surrounded by the nectary, and connected by the receptacle with the ovary. The ovary was shaped like a tiny vase, with a length of (6.8 ± 0.55) mm and a central diameter of (2.73 ± 0.44) mm. The placenta was a columnar structure extending to the bottom of the style. The ovules were arranged quite regularly along its surface in four vertical rows each containing about ~10–15 ovules. Papillae covering transmission tissue (TT) were located on opposite sides of the placenta. The stigma (4.32 ± 0.18), mm long, with developed papillae on the abaxial side, was connected with the ovary through the style, which was (2.88 ± 0.18) mm long and with a base diameter of (0.53 ± 0.08) mm (Figures 1A–G, 2A).

Examination of a cross-section of the base of the gynophore indicated the presence of both one gynophore vascular bundle (GV), with only one bundle of xylem in its center, and two dorsal carpellary traces (DC) on the outside of gynophore, which were amphicribral bundles (Figure 2A). The GV then differentiated into ventral and lateral carpellary traces (VC and LC), evidenced by the formation of many outer xylem bundles (Figures 2B, **7B**), and the dorsal carpellary trace transformed into the collateral vascular bundle by gradual inward movement of the xylem (Figures 2A–C).

**Figure 3:**
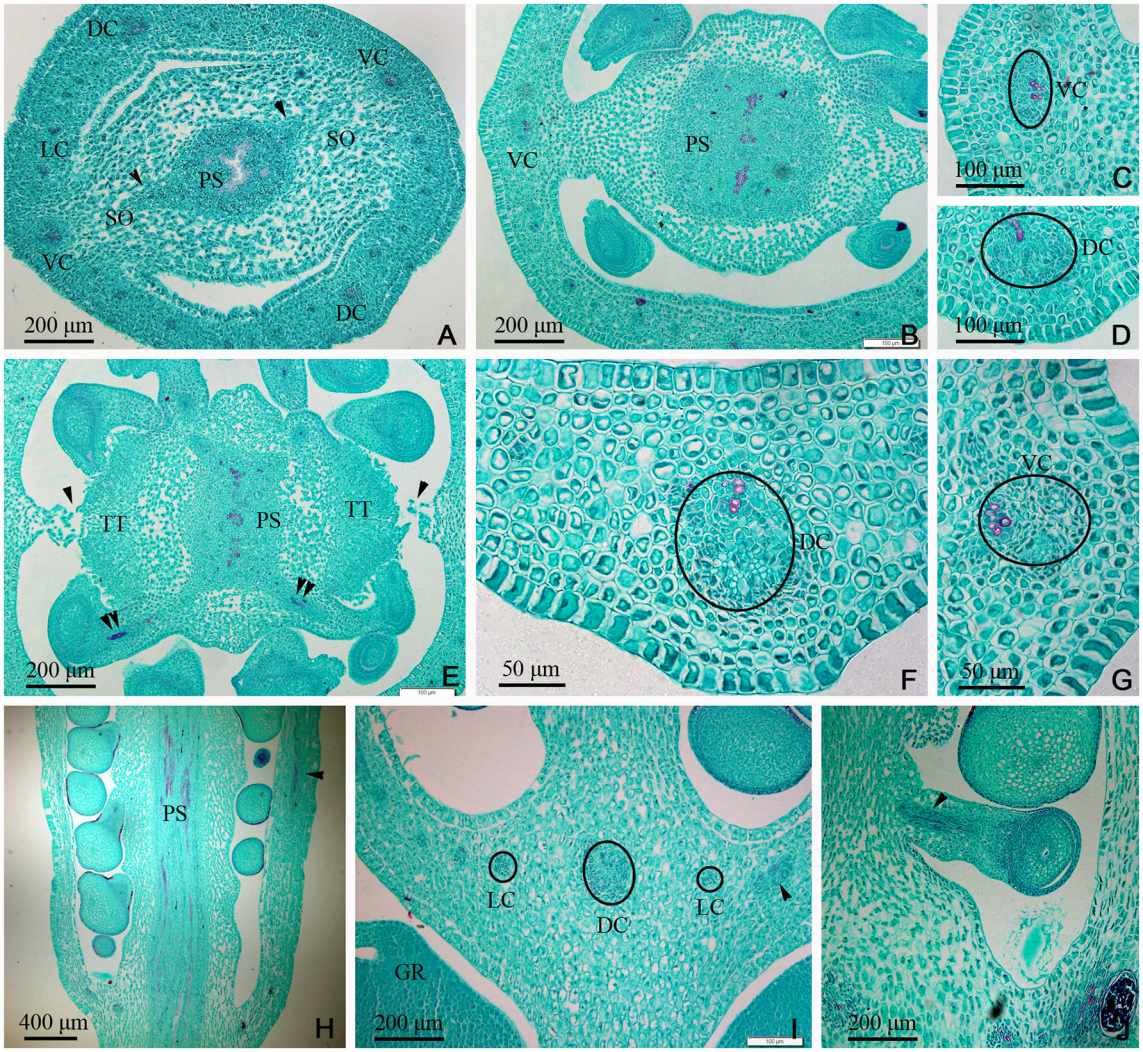
*Dianthus chinensis* L. gynobase sections. LC, lateral carpellary trace; PS, placental strand; SO, septum of the ovary; TT, transmission tissue; DC, dorsal carpellary trace; GR, glandular ring; VC, ventral carpellary trace. **(A)** A gynobase transection, showing an amphicribral placental strand extending outward along the wide ovary septum (arrow), and a collateral vascular bundle, including two ventral carpellary traces, two dorsal carpellary traces, and some lateral carpellary traces. **(B)** Transection a short distance above the level in **(A)**, showing the differentiating ovule provascular tissue and the narrow ovary septum. **(C,D)** Detailed views of local regions of ovary wall in **(B)**, showing a collateral ventral carpellary trace and a collateral lateral carpellary trace, respectively. **(E)** Transection a short distance above the level in **(B)**, showing amphicribral ovule traces (double arrows), the disappearing ovary septum (arrow), and the differentiated TT. **(F,G)** Detailed view of local regions of the ovary wall in **(E)**, showing collateral lateral carpellary trace and ventral carpellary trace, respectively. **(H)** Longitudinal section of a gynobase through the middle of the placenta, showing amphicribral ovule traces continually branching out of a placental strand and a collateral vascular bundle in the ovary wall (arrow). **(I)** Longitudinal section of a gynobase, not through the middle of the placenta, showing at least three collateral lateral, and one dorsal carpellary, traces visible in transverse and longitudinal sections (circle and arrow), respectively. **(J)** Longitudinal section of a local region of the gynobase, showing an amphicribral ovule trace in the funicle (arrow).

In the gynobase, the vascular bundles were mainly distributed in the placenta and ovary wall (carpel; Figures 3, **7C**). The placental strand (PS) and GV were continuous with one another, and then differentiated into numerous branches into the ovules (ovule trace, OT). Although, the PS form became irregular in the cross-section view, both the PS and the OT formed by its branches remained amphicribral (Figures 3A,B,H,J). The vascular bundles in the ovary wall, including the DC, VC, and LC, were all collateral (Figures 3C,D,F,G,I). In addition, no vascular bundle was found in the septum of the ovary (SO) in its gradual narrowing from the ovary wall and the TT appeared on the opposite side from the placenta (Figures 1C,E, 3A,B,E).

**Figure 4:**
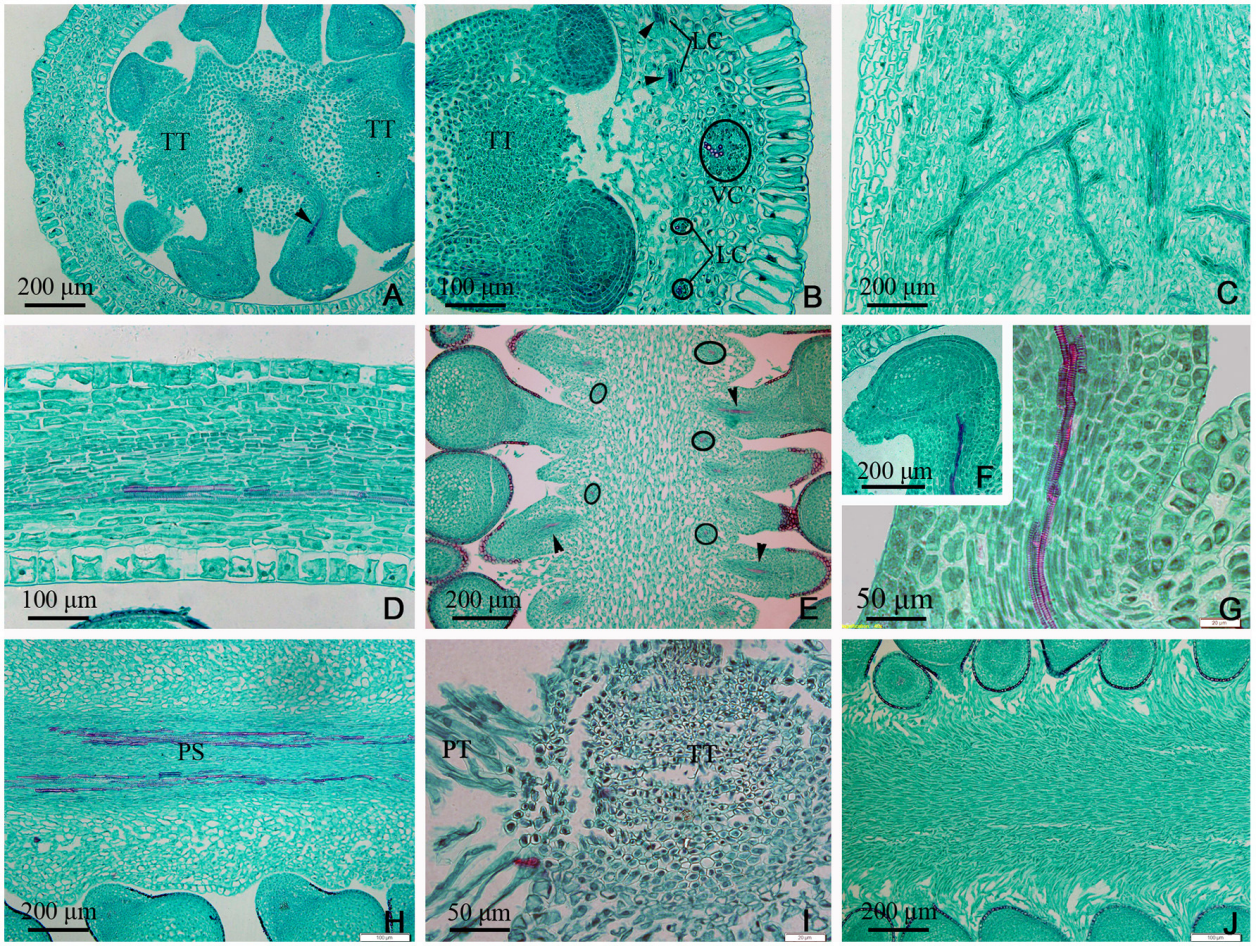
Sections through the middle of the ovary of *Dianthus chinensis* L. PT, papillae covering transmission tissue; TT, transmission tissue; LC, lateral carpellary trace; VC, ventral carpellary trace; PS, placental strand. **(A)** A transection through the middle of an ovary, showing the curved quadrilateral amphicribral placental strand, amphicribral ovule trace (arrow) deriving from a placental strand, and central placenta, separating from the ovary wall. **(B)** A local transection a short distance above the level in **(A)**, showing four collateral lateral carpellary traces in the ovary wall, visible in two longitudinal sections in the upper part (arrows) and in two transections in the lower part (small circles), 1–2 vessels within them, and a collateral ventral carpellary trace (large circle). **(C)** Longitudinal section of an ovary wall, showing the branching pattern of the vascular bundles. **(D)** Transection of an ovary wall, showing annular vessels in the xylem of a collateral vascular bundle. **(E)** Longitudinal section of the central placenta, showing transverse (circles) and longitudinal (arrows) amphicribral ovule traces. **(F)** Transection of an ovule, showing an amphicribral ovule trace in the funicle. **(G)** Detailed view of the ovule trace in **(F)**, showing annular vessels in the xylem of the amphicribral ovule trace. **(H)** Longitudinal section of the central placenta, showing the pattern of annular vessels in an amphicribral placental strand. **(I)** Detailed view of TT, showing small cells with dense cytoplasm and PT. **(J)** Longitudinal section, not through the middle of the central placenta, showing the distribution pattern of the papillae.

In the middle of the ovary, the PS cross-sectional area became smaller compared to that at the gynobase, and assumed a curved quadrilateral morphology (Figure 4A). Papillae covering transmission tissue (PT) developed (Figures 4A,B,I,J) and appeared to connect with the ovary wall, which is adjacent to the pollen tube extending down into the embryo sac. In the ovary wall, the DC, VC, and LC constantly branched to form an intertwined vascular network (Figures 4C, **7A**). However, the distribution, type, and structure of the vascular bundles in the middle of the ovary remained similar to that at its base (Figures 4A–J).

**Figure 5:**
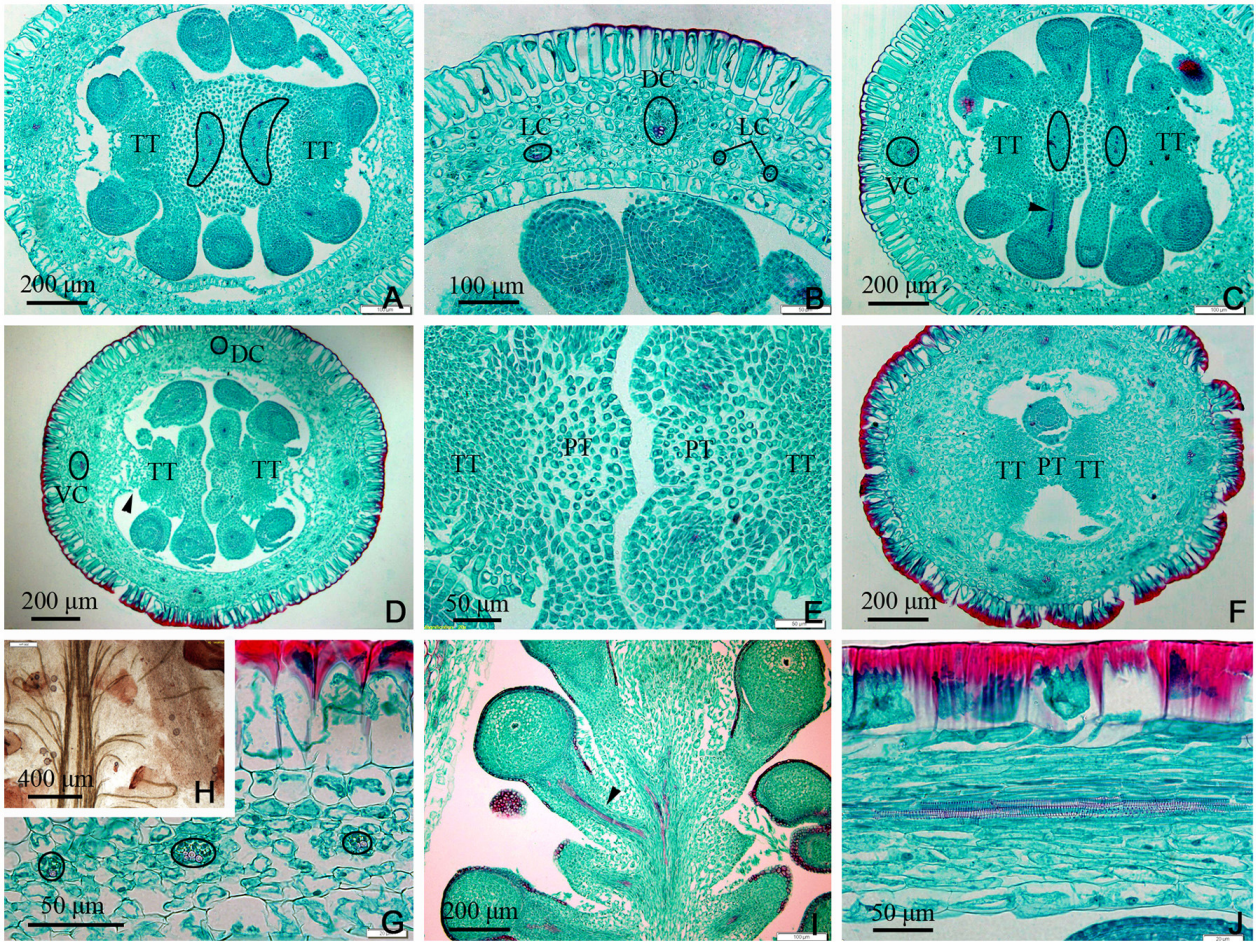
Sections of the upper part of an ovary of *Dianthus chinensis* L. PT, papillae covering transmission tissue; TT, transmission tissue; LC, lateral carpellary trace; DC, dorsal carpellary trace; VC, ventral carpellary trace. **(A)** Transection of an ovary shoulder, showing two excurved amphicribral placental strands. **(B)** Detailed view of a local area in **(A)**, showing one collateral dorsal carpellary trace and at least three collateral lateral carpellary traces. **(C)** Transection a short distance above the level shown in **(A)**, showing preliminary separation of the central placenta and an amphicribral placental strand. **(D)** Transection a short distance above the level shown in **(C)**, showing clear separation of the central placenta, disappearance of the placental strand, and reappearance of the septum (arrow). **(E)** Detailed view of a local area in **(D)**, showing the disappearance of vascular bundles in the left branch of the placenta and a small ovule trace in its right branch. **(F)** Transection of the upper part of an ovary below the junction between the ovary and styles, showing two locules separated by a septum mainly composed of TT and papillae, and complete disappearance of the placental strand. **(G)** Transection of an ovary wall, showing three collateral lateral ovary wall traces. **(H)** Detailed view of the upper central placenta, showing the branching pattern of the placental strand. **(I)** Longitudinal section of the upper part of an ovary, showing the branching pattern of the amphicribral ovule traces in the apical placenta. **(J)** Longitudinal section of an ovary wall, showing annular vessels in the xylem of a collateral bundle.

**Figure 6:**
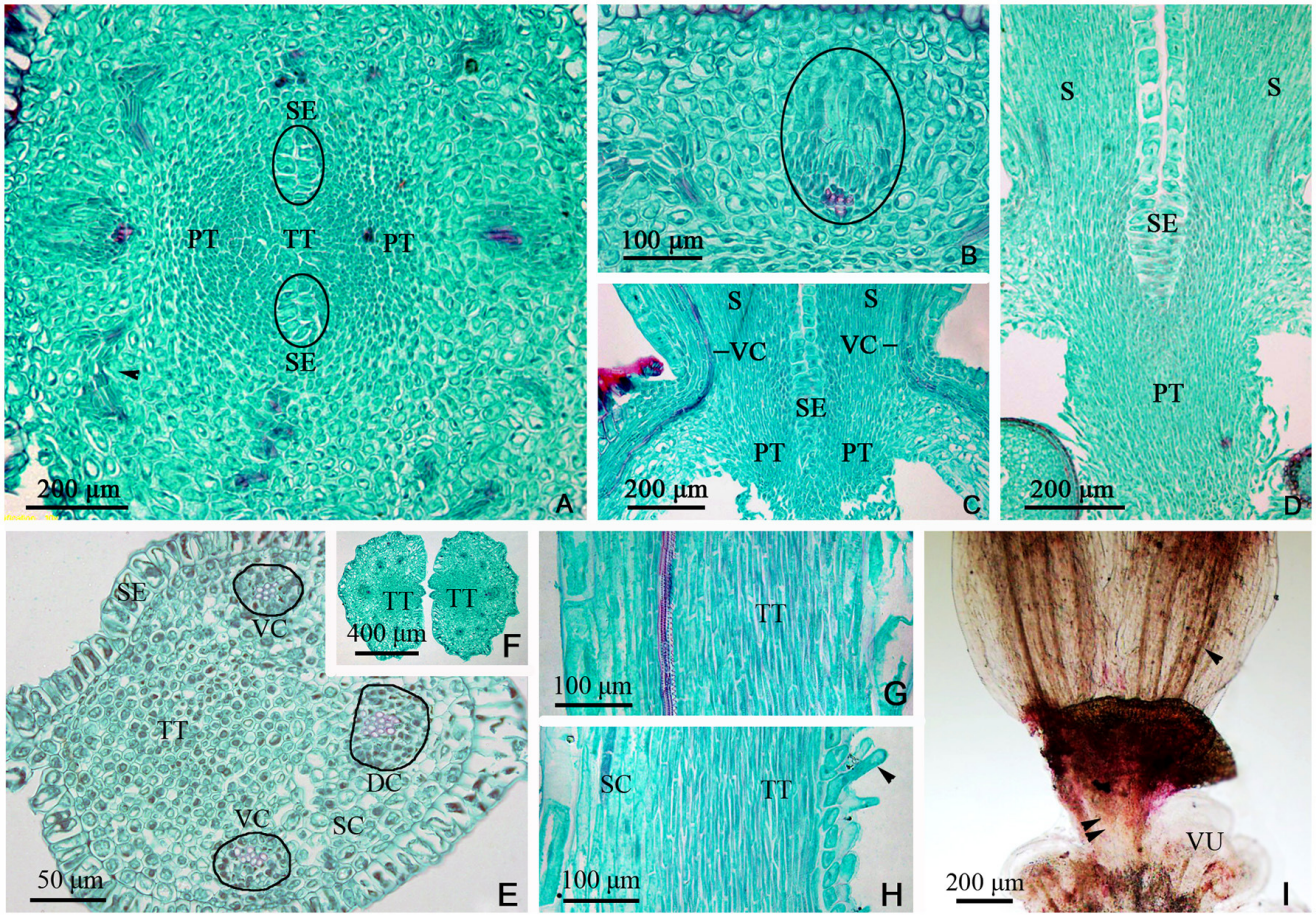
Sections and segregation images of the style and stigma of *Dianthus chinensis* L. SC, style/stigma cortex. SE, style epidermal cells; S, style; PT, papillae covering transmission tissue; TT, transmission tissue; DC, dorsal carpellary trace; VC, ventral carpellary trace; VU, ovule. **(A–D)** Style base. **(E–I)** Styles and stigma. **(A)** Transection of the juncture of an ovary and styles, showing the branching pattern of vascular bundles in the carpel (arrow), the lower end of the style, and placental tissue without vascular bundles. **(B)** Detailed view of a local area in **(A)**, showing a collateral ventral carpellary trace. **(C)** Longitudinal section through opposite ventral carpellary traces in two styles, showing the traces entering the styles from the ovary wall. **(D)** Longitudinal section, not through the middle of the styles, showing placental tissue participating in the styles. **(E)** Transections of the base of a style, showing two collateral ventral carpellary traces, one collateral dorsal carpellary trace, and the structure of the style. **(F)** Transection a short distance above the level shown in **(E)**, showing five vascular bundles in each style, suggesting that the ventral carpellary traces branch. **(G)** Longitudinal section of a stigma, showing annular vessels in the xylem of a collateral vascular bundle and TT. **(H)** Longitudinal section of a stigma between two adjacent vascular bundles, showing PT (arrow) and the structure of the style. **(I)** Segregation view of the juncture of an ovary and styles, showing the branching of the ventral carpellary trace (arrow) and the connection bands without vascular bundles.

**Figure 7:**
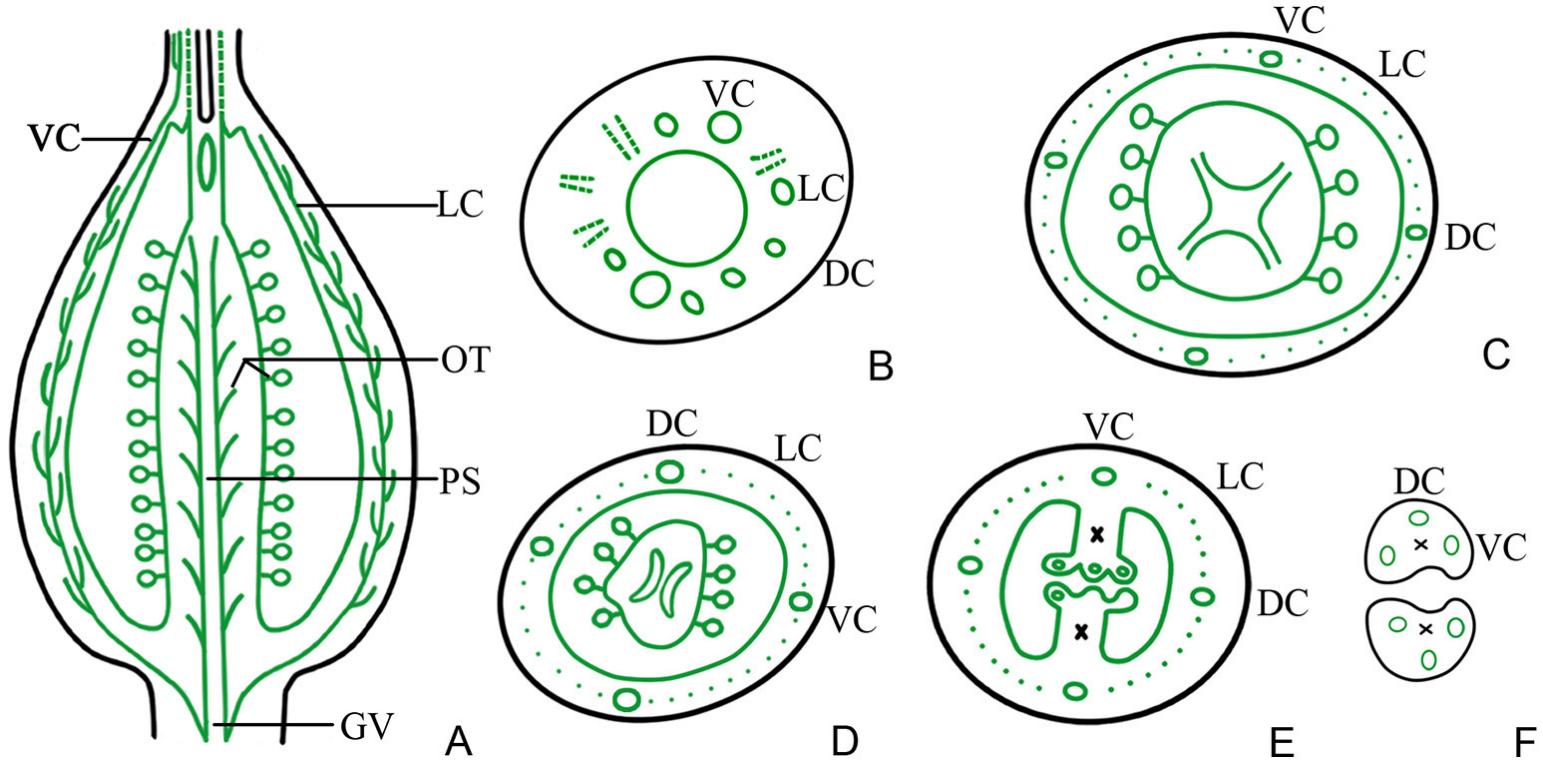
Diagram showing the distribution of vascular bundles in a *D. chinensis* L. pistil. DC, dorsal carpellary trace; VC, ventral carpellary trace; LC, lateral carpellary trace; PS, placental strand; GV, gynophore vascular bundle; OT, ovule trace. **(A)** Longitudinal section of a pistil, showing the arrangement of the primary vascular bundles. **(B)** Transverse section of a gynophore, showing the differentiation of DC, VC, and LC from GV. **(C)** Transverse section of a gynobase, showing OT from PS. **(D)** Vascular bundles in the upper part of the ovary, showing eyebrow-like bundles separated from the PS. **(E)** Vascular bundles in the upper part of an ovary, a short distance above the level shown in **(D)**, showing the division of the placenta and separation of the vascular bundle. **(F)** Transverse section of a style base, showing two VC and one DC in each style. The region of the TT in the style and placenta is indicated by an ×.

In the upper part of the ovary, the placenta split into two (Figures 5A,C–E, **7A,E**) and eventually appeared to be connected by papillae (Figure 5F). The PS split into three small bundles (Figures 5D,E, **7E**), from eyebrow-like (Figures 5A, **7D**) and oval tissues in succession (Figure 5C). There was no vascular bundle in the re-emerging SO at the apex of the ovary (Figures 5D,F, 6I, 7A). The vascular pattern in the upper part of the ovary was also similar to that in the middle and base of the ovary (Figures 5A–J).

The TT from the placenta extended upward to the style and stigma, forming their adaxial surface. The carpel also extended to the style and stigma, forming their abaxial tissue surface. Hence, the style and stigma comprised a composite structure, consisting of placenta and ovary wall; however, only the carpellary bundles entered the style and stigma (Figures 5F, 6C), and the PS terminated at the apex of the placenta (Figures 5E,F, 6A,I, 7A). Therefore, there were three vascular bundles in the style base, including two VC and one DC (Figures 6E, 7F). Subsequently, the VC branched once (Figures 6I, 7A), forming five vascular bundles in the upper style and stigma (Figure 6F), which were all collateral (Figures 6A–I).

## Discussion

In this investigation, we examined the structure of the different component tissues of the pistil of *D. chinensis* L. flowers at anthesis, and in particular the configuration of its vascular bundles. This study constitutes an essential first step to understand either the origin of pistil. Vascular bundles are amphicribral in the placenta (PS and OT) and collateral in the carpel (ovary wall in the strict sense; DC, CV, and LC).

The transformations of the vascular bundles in the transition zone between the root and the stem have been of interest for some years (Basconsuelo et al., 2002); however, little attention has been paid to those between the gynophore and the ovary wall. According to the results of this study, there is a transformation of the vascular system of the gynophore, resulting in an amphicribral bundle visible in cross-sections of the vascular system of the collateral bundle in the ovary wall (Figures 2A,B, 3C,D,F,G), and in longitudinal section the transition region is characterized by inward displacement of xylem, in which the xylem in the central part of the amphicribral bundles in the gynophore moves inward to the inner side of the vascular bundles in the ovary wall (Figure 2C). This transformation may be an important indication of the transition from an axial to a foliar (carpel, in the pistil) vascular system.

In *D. chinensis* flowers, the gynophore is located between the receptacle and the ovary, and the ovary is superior. Both amphicribral PS and collateral ovary wall vascular bundles are differentiated from GV (gynophore vascular bundle), while bundles in sepals, petals and stamens all branch from the receptacle. In other words, whether in structure or in position the PS is distinct from the vascular bundles in other parts of the flower. The connecting bands (CB) between the placental tissue and style are ruptured after fertilization, and the placenta free (Buell, 1952). Therefore, structurally, the placenta may be an independent unique organ without any connection with other parts of the ovary wall. This conclusion is consistent with the findings of Guo et al. (2013) and Wang et al. (2015).

In accordance with traditional opinion, the pistil of *D. chinensis* is composed of two fused carpels with a free central placenta and two separate styles. If this were correct, both structures and types of vascular bundles in PS should be the same as those in ovary wall; but our observations are inconsistent with this conjecture. In our observations, the PS and ovule trace are amphicribral while those in the ovary wall are collateral, implying the axial derivation for the placental bundles and contrarily a leafy precursor characteristics for the ovary (Figures 3–6) respectively. This inconformity between traditional opinions and the present result is not confined to *D. chinensis*. Numerous taxa of angiosperms have been reported to support amphicribral vascular bundles in placenta, including Papaveraceae (Kapoor, 1973, 1995), Leguminosae (Lersten and Don, 1966), Winteraceae (Tucker, 1975), Solanaceae (Dave et al., 1981; Wang et al., 2015), Gesneriaceae (Wang and Pan, 1998), Buxaceae (Von Balthazar and Endress, 2002), Annonaceae (Endress and Armstrong, 2011), Actinidiaceae (Guo et al., 2013), and Magnoliaceae (Liu et al., 2014; Wang et al., 2015), etc. These taxa cover the whole scope of angiosperms from the basal clade magnoliids to the terminal eudicot lineage in the phylogenetic tree (Angiosperm Phylogeny Group, 2009). Nevertheless, it is unfortunate that little attention has been thrown on this common disagreement although it has caused a lot of doubts to the traditional opinion.

The Unifying Theory interprets that the carpel (in classic sense) is a compound organ because it comprises not only an ovule-bearing shoot (placenta) but also a foliar part enclosing the shoot (Wang, 2010). According to the theory, the vascular bundles in the placenta should appear as radial symmetry (namely, amphicribral bundles), as is exactly observed in the placenta of *D. chinensis* in the present study, which provides strong evidence for it, and the gene expression of the placenta and ovary wall may be different. Actually, studies on gene expression patterns in flowers of model plants such as *Arabidopsis, Petunia*, and *Oryza*, indicate that (1) the expression of *STK* is required for normal development of the funiculus in *Arabidopsis*; (2) *FBP7* and *FBP11* are involved in proper ovule development in *Petunia*; (3) *AGL11* plays important roles in flower development; (4) *OsMADS13* functioning in flower organ differentiation and meristem determinacy in rice is restrictedly expressed in the placenta/ovules; (5) *DL* regulates carpel specification and midrib development in *Oryza sativa*; (6) *CRC* participates in carpel identity differentiation and carpel polarity establishment in *Arabidopsis* and also acts as a regulator in carpel development; and (7) *YABBY* is only expressed in the ovary wall (Angenent et al., 1995; Rounsley et al., 1995; Pinyopich et al., 2003; Yamaguchi et al., 2004; Dreni et al., 2007; Yoo et al., 2010; Li et al., 2011; Finet et al., 2016). All of the above genetic data indicate that there are differences in gene expression between the placenta and the ovary wall. From this we can see the origins of the two are different. The evidence of molecular biology and vascular structure imply that the placenta is a distinct floral organ which is equivalent to a secondary shoot and independent to the carpel, and that the placenta was recruited onto the ovary wall later in the evolution of angiosperms (Angenent et al., 1995; Roe et al., 1997; Skinner et al., 2004). In addition, the theory has also been supported by the evidences as follows: (1) In gymnosperms ovules are borne on fertile shoots so that are not able to give rise to carpels (Bierhorst, 1971; Biswas and Johri, 1997); (2) The ovule formation is completely independent to carpel in angiosperms mutants (Lersten and Don, 1966; Dave et al., 1981; Skinner et al., 2004); and (3) The seed is borne directly on an axis (shoot) in *Umkomasia mongolica* sp. nov. (Shi et al., 2016). So, overall, the current conclusion is reasonable that the angiosperm placenta is essentially an ovule-bearing branch with the carpel wall actually to be its subtending bract.

## Author contributions

X-MG planted and managed the experimental materials, designed the experiments, collected images, analyzed the data and wrote the article; Y-YY. carried out the preparation of paraffin-sections; LB performed the segregation experiment; R-FG supervised the experiments and complemented the writing.

### Conflict of interest statement

The authors declare that the research was conducted in the absence of any commercial or financial relationships that could be construed as a potential conflict of interest.
